# PP20 Health Technology Assessment To Sustain The Health Insurance Scheme: A Case Study On Anemia Treatment In Ghana

**DOI:** 10.1017/S0266462324001934

**Published:** 2025-01-07

**Authors:** Ivy Amankwah, Lieke Heupink, Ruby Aileen Mensah Annan, Brian Asare, Richmond Owusu, Emmanuella Abassah-Konadu, Hannah Asante, Lumbwe Chola, Katrine Frønsdal

## Abstract

**Introduction:**

In Ghana, the reimbursement of iron polymaltose complex (IPC) for iron-deficiency anemia treatment raises concerns as a potential cost driver for the National Health Insurance Scheme (NHIS). Prioritizing value for money is crucial for NHIS sustainability. With Ghana’s established health technology assessment (HTA) framework, we provide insights and preliminary findings in this case study on anemia treatment.

**Methods:**

To further support institutionalization, we follow the Ghana HTA process guidelines and Ghana HTA reference case. Simultaneously, to build wider capacity for HTA in Ghana, relatively new trained staff was engaged while anchoring the team at the HTA Secretariat from the Ministry of Health. We started a situational analysis, which includes desktop review and key-stakeholder interviews. An umbrella review was commenced simultaneously to identify systematic reviews assessing clinical effectiveness of IPC compared to other medicines on the essential medicine lists for all population groups. We will assess cost-effectiveness to ultimately inform coverage decisions and possible implications for organizational structures.

**Results:**

Anemia is a persistent public health issue in Ghana, impacting children under five, young women, and expectant mothers. The essential medicine list includes various treatments, but ambiguity exists in the standard treatment guidelines regarding when to administer IPC versus alternative medicines. Under the NHIS, the intravenous formulation of IPC stands out as the most expensive treatment. A systematic review identified three papers, focusing on infants, children, adults, and pregnant women. Preliminary findings suggest weak overall evidence supporting IPC’s superiority, although adults may exhibit higher tolerance for IPC compared to alternative treatments.

**Conclusions:**

Conducting an HTA on anemia treatments, despite the low treatment costs, is still significant as technical efficiencies can be achieved with high-volume drugs by analyzing prescription patterns and generating evidence to support potential changes. Findings from this HTA can be used elsewhere, especially in resource-constrained settings. Next steps involve finalizing the HTA and disseminating results to stakeholders.

